# Editorial: Surgical interventions in gastric cancer

**DOI:** 10.3389/fonc.2022.992965

**Published:** 2022-08-16

**Authors:** Ahmad Alromi, Giovanni Battista Levi Sandri, Luigi Bonavina, Sungsoo Park

**Affiliations:** ^1^ The Jordanian Ministry of Health, Departmant of General Surgery, Princes Hamzh Hospital, Amman, Jordan; ^2^ Department of Surgery, Korea University College of Medicine, Seoul, South Korea; ^3^ Department of Surgery, Ospedale Santa Scolastica, Cassino, Italy; ^4^ Division of General and Foregut Surgery, Department of Biomedical Sciences for Health, Policlinico San Donato, University of Milan, Milan, Italy

**Keywords:** gastric cancer, gastrectomy, laparoscopic gastrectomy, robotic surgery, surgery

Gastric cancer (GC) is the leading cause of cancer deaths and is considered the fifth most common cancer worldwide, accounting for 7.7% of all cancer deaths. Surgical treatment for GC has considerably improved in recent decades (1). The late nineteenth century marked the beginning of the surgical treatment of GC (2), and it is considered the only curative modality for early and some advanced forms of GC. (3–5)

The aim of surgical treatment of GC is to completely resect cancer cells with adequate margins and dissection of lymph nodes, followed by gastrointestinal reconstruction (6). Total, subtotal, and distal gastrectomies are routinely performed surgical procedures, and subsequent appropriate lymph node dissection is mandatory. The level of dissection is affected by the type of gastrectomy and disease extent.

Gastrointestinal reconstruction after gastrectomy can be achieved through various surgical techniques with permissible clinical outcomes as their main objectives. However, the ideal type of gastrectomy, level of lymph node dissection, and modality of reconstruction that can be chosen are still debated (7). Each functional reconstruction method has its advantages and disadvantages. Lee et al. showed that antiperistaltic reconstruction is significantly associated with delayed gastric emptying and bile reflux reduction.

According to the Japanese Gastric Cancer Treatment Guidelines 2018 (5th edition), gastrectomy surgeries are subdivided into curative and non-curative surgeries. Non-curative surgeries are offered to patients who are considered incurable and are divided into palliative and reduction surgeries depending on the aim of surgery (8). Palliative gastrectomy has not been approved by any guidelines to enhance the life span of patients with advanced GC and distant metastasis, except for critical circumstances or relieving symptoms (9).

The evolution of minimally invasive surgery is an important milestone in the field of surgical oncology (10). In early GC, the laparoscopic approach is favored in terms of the recovery period, risk of complications, and survival rate (5). Since Kitano et al. reported the first successful laparoscopic gastrectomy (LG) in 1994 (11), LG has globally replaced open gastrectomy in treating GC (11).

Tian et al. compared the outcomes, including efficacy and safety, of laparoscopic proximal gastrectomy (LPG) and laparoscopic total gastrectomy (LTG) in patients with proximal GC. They found that LPG can be an alternative to LTG for proximal GC, especially LPG with double-tract reconstruction/double flap technique (DFT), fewer lymph nodes were harvested, and the rate of postoperative anastomotic stenosis was higher in LPG than in LTG. Compared with LTG, LPG with esophagogastrostomy is associated with shorter operative time, less intraoperative blood loss, and higher rates of reflux esophagitis. No difference was found between LPG with double tract anastomosis/DFT and LTG.

In 2003, Hashizume et al. performed the first robotic gastrectomy (RG), which led to an increase in the number of studies on RG. Moreover, accumulating experience and the development and modification of robotic equipment led to the widespread application of RG in GC (12).

Feng et al. compared the perioperative and oncological outcomes of robotic and laparoscopic gastrectomy in patients with GC. They found that RG was associated with a longer operative time, less blood loss, earlier time to oral intake, shorter length of hospital stay, fewer complications, more retrieved lymph nodes, and higher cost.

In the last two decades, neoadjuvant chemotherapy (NACT) has played an essential role in improving the overall survival rate of patients with locally advanced disease (13–15). The elimination of micrometastases, improvement of tumor-related symptoms, and increase in survival rate are all advantages of NACT (14, 16). However, NACT may contribute to an increase in surgical complications and postoperative morbidities.

Regarding the optimal timing of surgery after NACT for locally advanced GC, Wang et al. concluded that patients who underwent surgery within 3–5 weeks experienced the maximal survival benefit without an increase in postoperative complications or a lower rate of complications.

Globally, the 5-year survival rate of GC is about 20%. The higher survival rates in South Korea and Japan are attributed to efficient screening programs that detect GC at earlier stages (17–19).

The effect of postoperative complications on the prognosis of GC remains controversial. Song et al. reported that serious complications after gastrectomy negatively affect the prognosis of patients with stage II/III GC. Serious complications worsen survival and are associated with inadequate adjuvant chemotherapy.

## Author contributions

SP supervised the conduct of this article. All authors approved the final version of the manuscript to be published.

## Conflict of interest

The authors declare that the article was conducted in the absence of any commercial or financial relationships that could be construed as a potential conflict of interest.

## Publisher’s note

All claims expressed in this article are solely those of the authors and do not necessarily represent those of their affiliated organizations, or those of the publisher, the editors and the reviewers. Any product that may be evaluated in this article, or claim that may be made by its manufacturer, is not guaranteed or endorsed by the publisher.
